# LncRNA HOTAIR promotes cell migration and invasion by regulating MKL1 via inhibition miR206 expression in HeLa cells

**DOI:** 10.1186/s12964-018-0216-3

**Published:** 2018-02-01

**Authors:** Peng Zheng, Ze Yin, Ying Wu, Yao Xu, Ying Luo, Tong-Cun Zhang

**Affiliations:** 1 College of Life Science and Healthy, Wuhan University of Science and technology, Wuhan, 430065 China; 2 Institute of Biology and Medicine, Wuhan University of Science and Technology, Wuhan, 430065 China; 3 Key Laboratory of Algal Biology, Institute of Hydrobiology, Chinese Academy of Sciences, Wuhan, 430072 China

**Keywords:** HOTAIR, MKL1, miR206, Migration, Invasion

## Abstract

**Background:**

Long non-coding RNAs (lncRNAs) have emerged as a new and crucial layer of gene regulation in recent years and regulate various biological processes such as carcinogenesis and metastasis. LncRNA HOTAIR, an oncogenic lncRNA, is involved in human tumorigenesis and dysregulated in cervical cancer. Megakaryoblastic leukemia 1 (MKL1), as a transcription coactivity factor, involved in cancer metastasis and cell differentiation. However, the precise mechanism of biological roles of HOTAIR and MKL1 in cancer cells remain unclear.

**Methods:**

The expression levels of HOTAIR and MKL1 were measured by quantitative PCR (qPCR), immunoblotting, in situ hybridization (ISH) and immunohistochemistry (IHC). Wound-healing and transwell assays were used to examine the invasive abilities of HeLa cells. Luciferase reporter assays and CHIP were used to determine how MKL1 regulates HOTAIR. Tissue microarray and immunohistochemical staining were used to assess the correlation between HOTAIR and MKL1 in Cervical cancer tissues in vivo.

**Result:**

In this study, we have identified that MKL1 had a role in the induction of migration and invasion in cervical cancer cells. Moreover, the expression level of MKL1, as the targeting gene of miR206, was decreased after HOTAIR inhibition in HeLa cells. Agreement with it, Highly level of MKL1 correlation with HOTAIR is validated in cervical cancer tissues. Importantly, HOTAIR is observed to participate in the silencing of miR206 expression. Interestingly, HOTAIR inhibition could also accelerate the expression of MKL1 in cytoplasm. What is more, MKL1 can activate the transcription of HOTAIR through binding the CArG box in the promoter of HOTAIR.

**Conclusion:**

These elucidates that the phenotypic effects of migration and invasion observed after HOTAIR inhibition, at least in part, through the regulation of MKL1 via inhibition of miR206 expression in HeLa cells. These data indicate the existence of a positive feedback loop between HOTAIR and MKL1. Together, these findings suggest that MKL1 is an important player in the functions of HOTAIR in the migration and invasion of cancer cells.

**Electronic supplementary material:**

The online version of this article (10.1186/s12964-018-0216-3) contains supplementary material, which is available to authorized users.

## Background

At least, 90% of the human genome is actively transcribed into non-coding RNAs (ncRNAs) [1], which are implicated in the regulation of multiple major biological processes impacting development, differentiation, and metabolism [2]. LncRNAs, which have gained widespread attention in recent years, are in general defined as mRNA-like, non-protein coding transcripts longer than 200 nucleotides and pervasively transcribed throughout eukaryotic genomes [3, 4]. Recent researches have revealed mechanisms involving lncRNAs in fundamental cellular processes, including apoptosis and cell cycle [5–7], chromatin modification [8, 9], genomic reprogramming [10, 11] and RNA processing [12]. What is more, they play critical roles in the progression of multiple diseases including cancer [13–15].

HOTAIR (Hox transcript antisense intergenic RNA) is one of the few well-studied lncRNAs, which located within the HOXC gene cluster on chromosome 12,with a length of 2158 nt [16, 17], and has emerged as a key regulator of carcinogenesis and metastasis and a potential prognostic marker [16, 18, 19]. Works pioneered by Howard Chang and colleagues has uncovered a compelling mechanistic basis for HOTAIR in cancer, which interacts with the polycomb repressive complex 2 (PRC2) to enhance H3K27 trimethylation, and decreases the expression of a large number of genes [20]. As a novel regulator in tumorigenesis, the expression of HOTAIR negatively correlates with tumor progression in several cancers, such as breast cancer [21–24], pancreatic cancer [25], colorectal cancer [26], gastric cancer [27, 28] and hepatocellular carcinoma [29, 30]. An increasing number of studies have focused on its biological function and its association with clinical prognosis in cancers, however the precise mechanism underlying its up-regulation remains largely unknown [31, 32].

As a member of the myocardin related transcription factor (MRTF) family, megakaryoblastic leukemia 1 (MKL1) is broadly expressed in many tissues [33] and functions as a co-activator of serum response factor (SRF), which played an important roles in the control of motile or contractile cell functions, especially during cancer metastasis, vascular smooth muscle cell and cardiac myocyte differentiation [33, 34]. MKL1 continuously shuttles between the nucleus and cytoplasm, and may help transducer signals from the cytoskeleton to the nucleus [35]. Rho signaling controls MKL1 activity by regulating the availability of G-actin, whose binding to the MKL1 amino-terminal RPLEL domain inhibits MKL1 nuclear import, promotes MKL1 nuclear export and inhibits activation by the MKL1/SRF complex [36, 37]. MKL1/SRF complex could bind to a serum response element, which contains a core sequence known as the CArG-box, This element was characterized by the sequence CC(A/T)_6_GG, and often found in the promoter regions of targeting genes.

In our previously work, we have successfully identified that the MKL1 expression level was significantly decreased after HOTAIR inhibition in HeLa cells [38]. However, the precise mechanism underlying the biological roles of HOTAIR and MKL1 was unknown. In this study, we analyzed the correlations and potential role of HOTAIR and MKL1 in the migration and invasion in HeLa cells.

## Methods

### Cell culture and siRNA transfection

Human cervical cancer cell line HeLa obtained from American Type Culture Collection were grown in DMEM (Hyclone, USA) at 37 °C with 5% CO_2_ in air containing 10% fetal bovine serum (FBS), 2 mM glutamine, 50 U/ml penicillin and 50 mg/ml streptomycin. For silencing the expression level of HOTAIR, 20 nM of siRNA targeted against HOTAIR (siHOTAIR-I or siHOTAIR-II) or a negative control siRNA (siNC) were transfected into HeLa cells using RNAi-mate (GenePharma, Shanghai, China). The RNA expression of HOTAIR was measured at 48 h after transfection by quantitative real-time PCR (qRT-PCR) as described below. The siRNA sequences were listed in Additional file 1: Table S1–1.

For MKL1 gene silencing, HeLa cells were transfected with 20 nM of siRNA targeted against MKL1 (siMKL1-I, siMKL1-II or siMKL1-III) and a negative control siRNA (siNC). Transfection with 20 nM siMKL1 or siNC was performed using RNAi-mate (GenePharma, China). Cells were harvested at 48 h after transfection and MKL1 gene knockdown was assessed by Western blotting. All the siRNAs were purchased from GenePharma Co. Ltd. The siRNA sequences were listed in Additional file 1: Table S1–1.

### RNA isolation and *qRT-PCR*

Total RNA was extracted from cultured cells using Trizol Reagent (Invitrogen) according to the manufacturer’s protocol. A Nanodrop 2000 spectrophotometer (Thermo Scientific) was used to measure the concentration of total RNA. RNA was then reverse transcribed into first strand cDNA with Revert Aid First Strand cDNA Synthesis Kit (Ferments, USA).And quantitative PCR was carried out using the SYBR Green PCR Master Mix (Roche) and Light Cycler 480 Real-Time PCR system (Roche). The GAPDH gene was used as the endogenous control gene for normalizing expression of the target genes. Each sample was analyzed in triplicate. The thermo cycling program consisted of one hold at 95 °C for 5 min, followed by 40 cycles of 10 s at 95 °C, 30 s at 60 °C and 30 s at 72 °C. Melting-curve data were then collected to verify PCR specificity and the absence of primer dimers. The primers sequences were listed in Additional file 1: Table S1–2.

MiRNA was isolated by the mirVana miRNA isolation Kit (Ambion) according to the manufacturer’s protocol*.* A NanoDrop 2000 spectrophotometer was used to measure the concentration of total miRNA. Quantitative analysis of miR-206 expression was assayed using a Hairpin-it miRNA real-time PCR Quantitation Kit (GenePharma, Shanghai, China). The small nuclear RNA U6 was used as an internal control. Each sample was analyzed in triplicate. All primers sequences were listed in Additional file 1: Table S1–2.

### Isolation of nuclear and cytoplasmic extract

The nuclear extraction was prepared using an NE-PER Nuclear Cytoplasmic Extraction Reagent kit (Pierce, Rockford, IL, USA) according to the manufacturer’s instruction. In briefly, cells were washed twice with cold PBS and centrifuged at 500 g for 5 min. The cell pellet was suspended in 200 μl of cytoplasmic extraction reagent I. Then, vortex the tube vigorously on the highest setting for 15 s. The suspension was incubated on ice for 10 min followed by the addition of 11 μl CER II, vortexed for 5 s, incubated on ice for 1 min and centrifuged for 5 min at 16000 g. The supernatant (cytoplasmic extract) was immediately transferred to a clean pre-chilled tube. The insoluble pellet fraction, which contains crude nuclei, was resuspended in 100 μl of nuclear extraction reagent by vortexed during 15 s and incubated on ice for 10 min, then centrifuged for 10 min at 16000 g. The supernatant (nuclear extract) was immediately transferred to a clean pre-chilled tube and used for the subsequent experiments.

### Plasmid constructs and expression

The full-length MKL1 gene (accession number CR456522.1) cDNA was amplified by RT-PCR from total RNA isolated from HeLa cells, and inserted into the cloning vector pMD-18 T (TaKaRa, USA). And the sequences of PF-1 and PR-1 for amplifying MKL1 were listed in Additional file 1: Table S1–3. Each construct was verified by DNA sequencing (Invitrogen, USA). The primers PF-2 and PR-2 were used to amplify the coding regions of MKL1 from pMD-18 T-MKL1. The fragment was cloned into the mammalian expression vector pCDNA3.1/myc-his B (Invitrogen, USA). The BamH I and XhoI restriction sites were designed in the forward and reverse primers respectively. All the sequences of primers were listed in Additional file 1: Table S1–3.

For expressing MKL1, the plasmid pCDNA-MKL1 was transfected into HeLa cells by transfection reagent Lipofection 2000 (Invitrogen) according to the manufacturer’s instructions, after the cells were cultured in serum-free medium without antibiotics at 60% confluence for 24 h. After incubation for 6 h, the medium was removed and replaced with normal culture medium for 48 h. And the plasmid pCDNA3.1/myc-his B was used as the negative control. The expression of MKL1 was assessed by Western blotting. GAPDH was used as a loading control.

### Generation of MKL1 KO cells by CRISPR/ cas9 technology

As a powerful and useful genome editing tool, a paired-guide RNA CRISPR–Cas9 library [39, 40] was used to construct MKL1 KO stably genetic cells by deleting a large genomic fragment of MKL1 to investigate its function. Plasmid CP-C9NU-01 carried fluorescent protein mCherry and resistance gene Neo, which expressed an RNA-guided DNA endonuclease cas9 to cleave DNA. And the sgRNA expression vector pCRISPR-SG01 was carried resistance gene Hygro. All plasmids were purchased from Gene Copoeia. Four sgRNA targeting interesting gene MKL1 were designed. The sequences of the target MKL1-gRNA are listed in Additional file 1: Table S1–4. Then, we enumerated all possible pgRNAs according to previously reported [41]. The plasmidCP-C9NU-01was co-transfected into HeLa cells with the pgRNAs plasmids. After 48 h, cells were selected with neomycin and hygromycin B resistances for 3 weeks, until one clone was selected from CP-C9NU-01/pCRISPR-SG01-pgRNAs transfected HeLa cells (defined as MKL1-KO) or CP-C9NU-01 transfected HeLa cells. The expression level of MKL1 was determined by western blotting.

### Wound healing assay

Cells were seeded into a 6-well plate and allowed to grow to 70% confluences in complete medium. Cell monolayers were wounded by a plastic tip (1 mm) that touched the plate. Then wash the cells with PBS to remove the debris. The cells were transfected and incubated for 24 h. Cells migrating into wound surface and the average distance of migrating cells was determined under an inverted microscope at designated time points.

### Cell invasion assay

Transwell chambers (Corning, 8.0 μm pore size) coated with Matrigel (BD Biosciences, USA) were used to measure the invasiveness of cancer cells. In brief, 2 × 10^5^ cells were plated in the upper chamber in serum-free media. And the bottom chamber was covered with media with 10% FBS. After 48 h incubation, the bottom of the chamber insert was fixed in methanol for 15 min and stained with Giemsa stain. Invading cells were photographed and counted on the stained membrane under microscope. Each membrane was divided into four quadrants and an average from four quadrants was calculated.

### Western blotting

Protein extracts (10 μg) prepared with RIPA lysis buffer were resolved on a 12% SDS-PAGE gel, and transferred to an Immobilon-P PVDF transfer membrane (Millipore, Bedford, MA) by electro-blotting. After blocking with 5% non-fat milk, membranes were incubated overnight with a 1:1000 dilution of antibodies at 4 °C. Blots were then incubated with peroxidase-conjugated anti-mouse or anti-rabbit IgG (KPL, Gaithersburg, MD) for 1 h at RT at a 1:1000 dilution and then developed using a Super Signal West Pico kit (Pierce Biotechnology). Immunoblots were scanned using an Image Scanner (GE healthcare). Blot densitometry analysis was performed using Image J (National Institutes of Health).The following antibodies were used for Western blot analyses: rabbit anti-MKL1 polyclonal (Abcam), mouse anti-GAPDH polyclonal (Sigma).All the analysis was performed in triplicate.

### Bioinformatical analysis

The potential microRNA binding sites of MKL1 predicted by computer-aided algorithms were obtained from microRNA.org-target program (http://www.microrna.org/microrna/home.do), TargetScan Human (Release 7.0) (http://www.targetscan.org/vert_61/), and StarBase v2.0 (http://starbase.sysu.edu.cn/in-dex.php).

### Assay of luciferase activity

Site-directly mutant was used to change the binding sites of miR-206 in the 3’UTR of MKL1. To perform luciferase activity assays, the miR-206 binding sites from 3’ UTR of MKL1 or mutant 3’ UTR were cloned into the pGL3 reporter luciferase vector (Merck), which a 1075-bp fragment (nt3147–4221) 3’UTR of MKL1 identification number NM_001318139.1. We named these plasmids as pGL3-MKL1–3’UTR, pGL3-MKL1–3’UTR-mut. The sequences of primers were listed in Additional file 1: Table S1–5. For reporter assay, 20 nM miR-206 mimics or control miRNA was co-tranfected with 0.1 μg of the pGL3-MKL1–3’UTR or pGL3-MKL1–3’UTR-mut into HeLa cells using lipofectamine 2000 (Invitrogen)*.* Luciferase activity was measured 48 h after transfected as described previously.

The sequences of HOTAIR promoter were amplified from genome DNA extracted from HeLa cells. Then, the fragment was cloned into the pGL3 reporter luciferase vector (Merck). The BamH I and XhoI restriction sites were designed into the forward and reverse primers respectively. All the sequences of primers were listed in Additional file 1: Table S1–6. The primers HOTAIR promoter △PF/△PR and mutant PF/PR were used to delete or mutant the CArG box sequences in HOTAIR promoter region. We named these plasmids as pGL3-HOTAIR, pGL3-HOTAIR-mut and pGL3-HOTAIR-△. For reporter assay, 0.2 μg pCDNA-MKL1 or empty control plasmid pCDNA3.1 was co-transfected with 0.8 μg of the pGL3-HOTAIR promoter, or pGL3-HOTAIR-mut or pGL3-HOTAIR-△ using lipofectamine 2000 (Invitrogen) into COS7 cells. For all report Luciferase assay, the cells were harvested at 48 h after post-transfection using lysis buffer. The activities of luciferase in cell lysates were determined using the Dual-Luciferase Reporter Assay System (Promega).

### Chromatin immunoprecipitation (ChIP) analysis

The ChIP assay was performed using Chromatin Immunoprecipitation (ChIP) Assay Kit (Millipore), following manufacturer’s instructions. Briefly, 1 × 10^7^ cells were cross-linked with 1% formaldehyde for 10 min at 37 °C. Then, cells were scraped and resuspended with ice-cold 1 × PBS with protease inhibitor cocktails (Pierce). Cells were then lysed and sonicated to shear DNA to an average length between 200 and 1000 base pairs (bp). All the procedure should keep samples on ice. Lysates were immunoprecipitated with anti-MKL1 (Abcam) at 4 °C for overnight. Immunoprecipitation with irrelevant normal IgG was used as a negative control. Immune complexes were isolated with Protein A/G Sepharose beads at 4 °C for 1 h. After washing, DNA fragments contained in immune complexes were purified, and amplified by PCR reactions. Sequences of primer pairs used for ChIP assay of MKL1 binding to HOTAIR promoter are shown in Additional file 1: Table S1–7.

### Rna fish

RNA FISH was performed with modification of published techniques [42, 43]. All reagents were RNase-free. Cells were grown on sterile glass bottom culture dish. The culture dishes were washed with PBS three times and then fixed by 4% paraformaldehyde for 30 min at RT. Cells were permeabilized with 0.1% Triton X-100 in PBS for 5 min at 4 °C and rehydrated in a solution containing 2 × SSC and 50 formamide for 5 min at RT. Then each coverslip of rehydrated cells was incubated at 37 °C at least 1 h in 40 μl of hybridization solution (2 × SSC (25% *v*/v), deionized formamide (10% v/v), dextran sulfate, 2 mM vanadylribo nucleotide complex, 0.002 mg/mL nuclease-free bovine serum albumin, 1 mg/mL E.colit RNA and 250 μl/mL of N-50 DNA). Coverslips are placed cell-side-down on this solution in a humidified chamber for incubation. The cells were incubated at 65 °C for m5 min in 40 μl of hybridization solution containing 250 ng probes. And then the coverslips are incubated at 37 °C overnight (or 42 °C for 4–5 h) in a humidified chamber. The cells were then washed with wash buffer (50% SSC, 50% deionized formamide), and stained with DAPI (4′6-diamidino-2-phenylindole) for 10 min at RT. Subsequently, cells were washed three times with wash buffer thoroughly and examined using a LSM 710 laser scanning confocal microscope (Carl Zeiss, Germany).

The probes were designed by the website of Biosearchtech (https://www.biosearchtech.com/support/tools/design-software/stellaris-probe-designer). Ten targeted probes against HOTAIR were synthesized, which labeled with the fluorophore Alexa Fluor 647 at the 5 terminal. And the probe of miR-206 was purchased from Invitrogen. The detail sequences were shown in Additional file 1: Table S1–8 & Additional file 1: Table S1–9.

### Hematoxylin and eosin (HE) staining

Paraffin embedded cervical cancer was sliced, rehydrated through an xylene and ETOH series, and then stained with Hematoxylin (Gill’s 1X) for 5 min. Rinse slides in running tap water for 5 min and dunk in Acid Alcohol (1%HCl in 70% ETOH) 2–3 times until the sections turn pink. Rinse slides in tap water again for 3–5 min and 5–6 slow dunks in ammonia Water (1 mL NH_4_OH in 1 L H_2_O). Rinse slides 3–5 min in tap water and then counter stain with Eosin Y solution for 1 min. Dehydrate through ETOH series and clear in xylene series. Coverslip slides were mounted in ProLong Gold (Invitrogen, Carlsbad, CA) and left overnight at room temperature.

### Immunofluorescence staining, imaging, and computational analysis

Cells were grown on sterile glass bottom culture dish. Cells were fixed in 4% formaldehyde for 30 min and permeabilized in 1% FBS, 0.2% Triton-X100 on ice for 5 min. After washing, cells were blocking with 1% BSA for 1 h at RT and incubated with rabbit anti-MKL1 polyclonal at a dilution of 1:200 overnight. Then, cells were incubated with Dylight 488-conjugated Goat anti-Rabbit IgG (H + L) (Beyotime, China) at a dilution of 1:100 for 2 h in dark. Cells were stained with 4′6-diamidino-2-phenylindole (DAPI) for 15 min. Subsequently, cells were washed three times with 1 × PBS thoroughly and examined using a LSM 710 laser scanning confocal microscope (Carl Zeiss, Germany).

### Tissue microarray and immunohistochemical staining

A Cervical squamous cell carcinoma microarray was purchased from National Human Genetic Resources Sharing Service Platform (Shanghai, 2005DKA21300), which contains 31 cancers and 31 adjacent non-cancers. All samples were deparaffinized, rehydrated through graded alcohol, washed with PBS. Samples were blocked with 2% BSA for 1 h at RT and incubated with rabbit anti-MKL1 antibodies diluted 1:200 for 4 °C overnight. The corresponding secondary antibody was used for 1 h at 37 °C. Then, slides were stained with 4′6-diamidino-2-phenylindole (DAPI) for 15 min.

### Statistical analysis

Statistical analysis was done using SPSS standard version 13.0 software. The independent Student’s t-test was used to compare the continuous variables between two groups. The data were expressed as means ± SD from at least three independent determinations. Values of *P* < 0.05 (or *P* < 0.01) were considered statistically significant.

## Results

### Knockdown of HOTAIR decreased the expression of MKL1

**Fig. 1:**
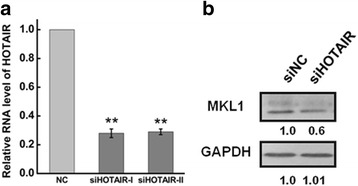
Western blotting determine MKL1 expression. a HeLa cells were transfected with siHOTAIR or siNC for 48 h. The efficiency of HOTAIR knockdown was determined by qRT-PCR. The expression level of HOTAIR was normalized to GAPDH. b Western blots analysis of the MKL1 protein expression at 48 h after transfected with siHOTAIR (siHOTAIR-I and siHOTAIR-II) or siNC. GAPDH was used as an internal control. Data are presented as means ± S.D. and results are from one representative experiment of at least three. *, *P* < 0.05; **, *P* < 0.01 (Student’s t test)

HOTAIR, as a negative prognostic factor, has been reported that it correlated with cancer cell apoptosis, invasion and metastasis [44–46] in several cancer cell lines [15, 47, 48]. We have uncovered that HOTAIR may involve in diverse biological processes by mediating proteins expression level [49]. Then, western blotting was used to analyze MKL1 expression after HOTAIR inhibition. As shown in Fig. 1a & b, MKL1 expression was obviously decreased after transfection with siRNA against HOTAIR.

### MKL1 contributes to the effects of the migration and invasion of HOTAIR knockdown

**Fig. 2:**
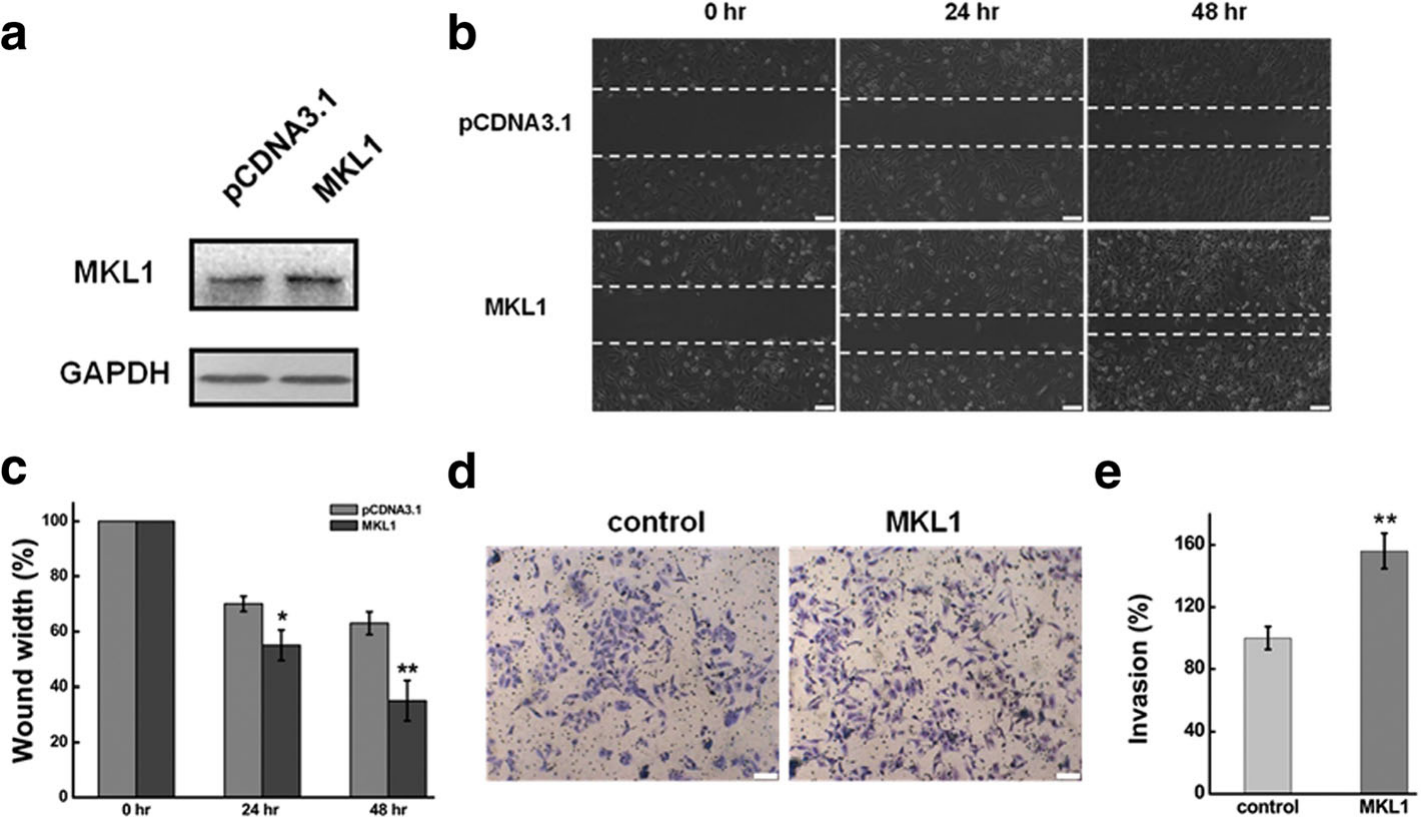
Overexpression MKL1 promoted cell migration and invasion in HeLa cells.**a** HeLa cells were transfected with pMKL1 or pCDNA3.1. The MKL1 expression level was determined by western blot at 24 h and 48 h after transfection. pCDNA3.1 serves as the negative control and GAPDH serves as the loading control. **b** The effect of MKL1 overexpression on cell migration was determined by wound healing assay. **c** Quantification of the wound healing assay. **d** The effect of MKL1 overexpression on cell invasion was determined in a Boyden chamber assay. **e** The number of cells on the underside of the filter was determined and significantly (*P <* 0.05) changed invasion is indicated. Data are presented as means ± S.D. and results are from one representative experiment of at least three. *, *P <* 0.05; **, *P <* 0.01 (Student’s t test)

As a important transcription factor, MKL1 functions as an organizer of a number of critical involved in signals transduction [35], differentiation [50], apoptosis [51], and cell mobility [52]. The role of MKL1 in HeLa cells was evaluated by silencing/overexpression of MKL1 using transient transfection of validated siRNAs and cDNAs. The plasmid pCDNA3.1-MKL1 was used to express MKL1 in HeLa cells. And pCDNA3.1 was as the negative control. GAPDH serves as the loading control. Western blotting was performed to determine MKL1 expression at 48 h after transfection. As shown in Fig. 2a, MKL1 was significantly increased in HeLa cell after transfected with plasmid pCDNA3.1-MKL1.

The migration and invasiveness capabilities of HeLa cells after MKL1 overexpression were evaluated by wound-healing assay and matrigel invasion assay. The wound-healing assay demonstrated that overexpression MKL1 may significantly induce the cell migration in HeLa cells compared with negative control (Fig. 2b). We found that the percentages of origin wound width was significantly decreased (approximately 30%, *P < 0.01*) in comparison with the negative control at 24 h and 48 h after transfected with pCDNA3.1 (Fig. 2c). As shown in Fig. 2d & e, the number of invaded cells increased significantly in MKL1 overexpression cells in comparison with the negative control.

**Fig. 3:**
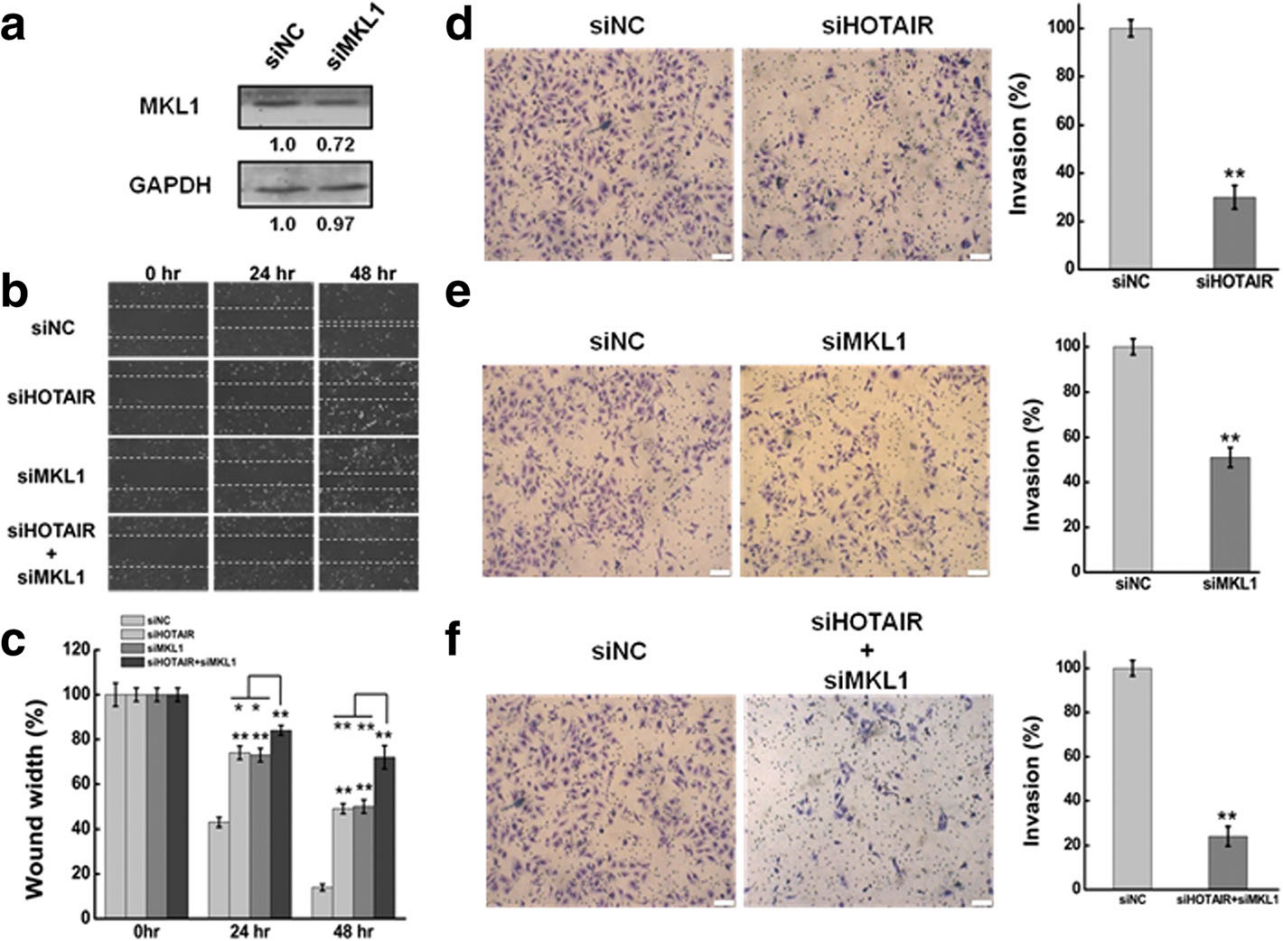
**a** Western blots analysis of the MKL1 protein expression at 48 h after transfected with siMKL1 (siMKL1-I, siMKL1-II and siMKL1-III) or siNC. GAPDH was used as an internal control. **b** The effect of knockdown HOTAIR or/and MKL1 on cell migration was determined by wound healing assay. **c** Quantification of the wound healing assay. **d, e, f** The effect of HOTAIR or/and MKL1 inhibition on cell invasion was determined in a Boyden chamber assay. And the number of cells on the underside of the filter was determined and significantly (*P* < 0.05) changed invasion is indicated. Data are presented as means ± S.D. and represent results from three independent experiments. Statistically significant differences are indicated: *, *P <* 0.05; **, *P <* 0.01 (Student’s t test)

RNA interference (RNAi) technology was used to silence the expression of MKL1. And western blotting analysis was used to detect the knockdown efficiency. The MKL1 expression was significantly decreased at 48 h after transfected with siRNA against with MKL1 (Fig. 3a). The wound-healing assay that MKL1 inhibition resulted in a significantly decreased of cell migration in HeLa cells compared with negative control (Fig. 3b & c). Meanwhile, the number of invaded cells decreased significantly in MKL1 knockdown cells in comparison with the negative control using a Boyden chamber assay (Fig. 3e).

**Fig. 4:**
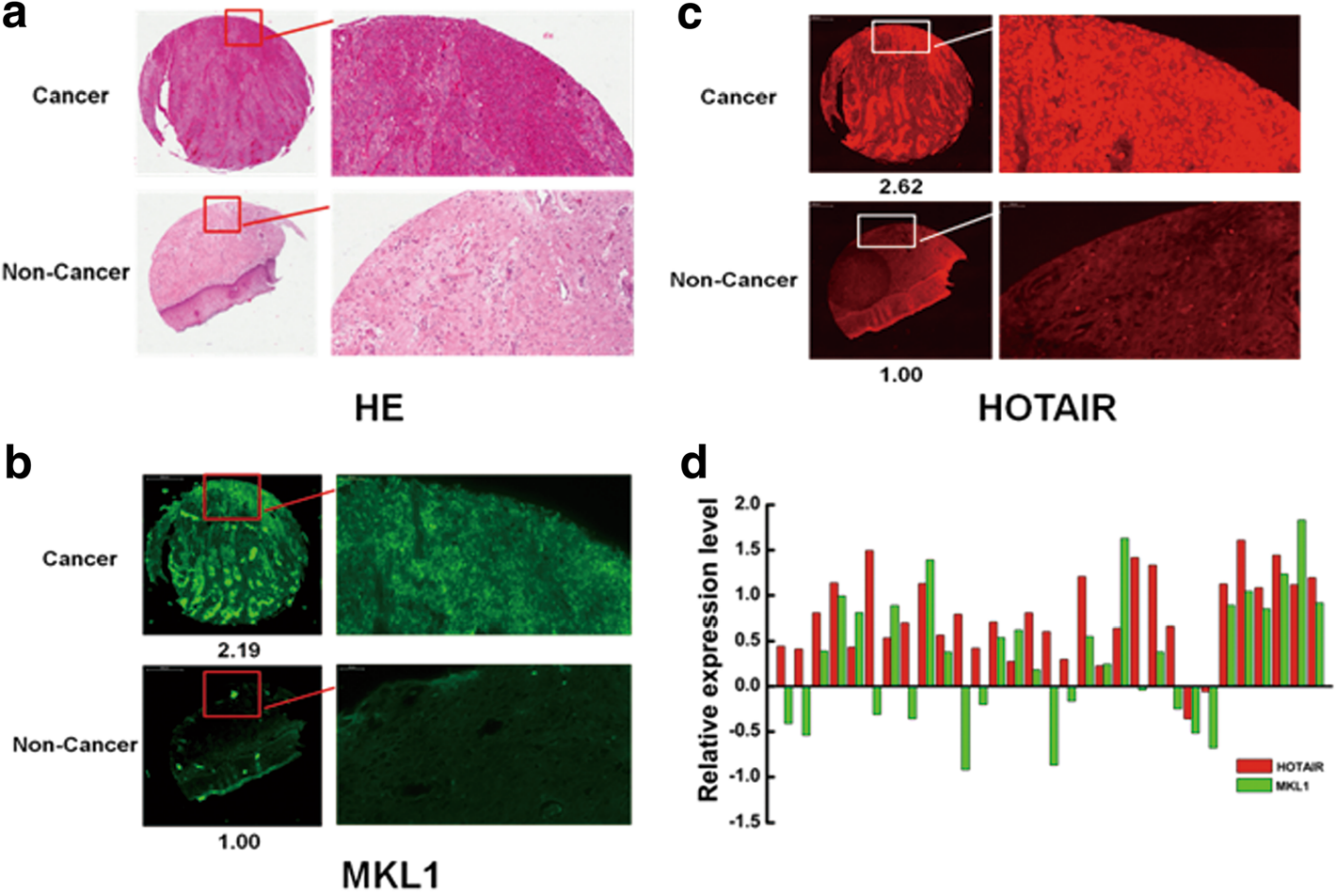
High level of MKL1 significantly correlate with HOTAIR expression in Cervical cancer patient. **a** Hematoxylin and eosin (HE)-stained cervical cancer tissues and adjacent normal tissues. **b** HOTAIR stained by in situ hybridization; high level of HOTAIR expression in cervical cancer tissues compared with adjacent normal tissues. **c** The expression of MKL1 was detected in cervical cancer tissues and adjacent normal tissues by IHC. The MKL1 expression was enhanced in cervical cancer tissues compared with adjacent normal tissues. The images were acquired by Pannoramic MIDI with a 20 × microscope objective. **d** The correlation between the expression of HOTAIR and MKL1 in cervical cancer tissues from 31 patients was shown. Red represented the RNA expression of HOTAIR. And Green represented MKL1 protein expression level

To further confirm the function effects of MKL1 on the migration and invasion in HeLa cells, we deleted MKL1 expression using CRISPR/cas9 technology. As shown in Additional file 2: Figure S1A, the protein abundance of MKL1 was significantly decreased 90% in MKL1_KO_1 cells compared with control. However, the expression of MKL1 was almost not measured in MKL1_KO_2 cells, which was used to study the functions effect of MKL1. Furthermore, the invasiveness and migration capacity were significantly decreased in the MKL1-KO_2 cells, as shown in Additional file 2: Figs. S1B, 1C, 1D, 1E. These results were consistent with that in transiently transfected cells. These result revealed that MKL1 can increased the ability of migration and invasiveness in HeLa cells.

To investigate whether MKL1 has a role in mediating the functions of HOTAIR and explore the molecular sequelae of MKL1 inhibition/overexpression, we co-transfection with siRNA of HOTAIR and MKL1 to determine the migration and invasion of HeLa cells. As shown in Fig. 3b & c, the migration was drastically decreased after HOTAIR or MKL1 knockdown. Notably, co-transfection of HeLa cells with siRNA of HOTAIR and MKL1 further suppressed the effect compared to silence HOTAIR or MKL1. Meanwhile, the invasive capability of HeLa cells was significantly reduced approximately 65% and 50% in comparison with the negative control after transcfection 48 h with siHOTAIR or siMKL1 **(**Fig. 3d & e). However, the invasion was more decreased after co-transfection with siRNA of HOTAIR and MKL1 **(**Fig. 3f).

To further confirm the correlation of HOTAIR and MKL1 and their biological significance in cervical cancer patient, we detected the expression level of HOTAIR and MKL1 in a TMA II containing 31 informative patients with cervical cancer tissues and their corresponding adjacent non-cancer tissues. Immunofluorescence analysis was used to analyze the expression of HOTAIR in cervical cancer tissues. As shown in Fig. 4b & Additional file 3: Figure S2, we found that HOTAIR was predominantly higher expressed in cancer tissues than adjacent non-cancer tissues. While MKL1 nearly showed the same expression trend with HOTAIR in the cancer tissues. Immunohistochemical staining showed that cervical cancer tissues, but not their adjacent non-cancer tissues, had high expression of MKL1 (Fig. 4c & Additional file 3: Figure S2). Among 31 patients’ tumor tissue samples tested, about 19 (61.2%) patients showed high expression of both HOTAIR and MKL1 **(**Fig. 4d). This uncovered that high level of MKL1 significantly correlated with HOTAIR expression in cervical cancer patient. The in vivo data complemented the functional in vitro studies of HOTAIR and MKL1.

Based on these results, we propose that the biological effects of HOTAIR are at least in partially mediated by regulation of MKL1 expression on the migration and invasiveness in HeLa cells.

### MKL1 is a downstream target of miR-206

**Fig. 5:**
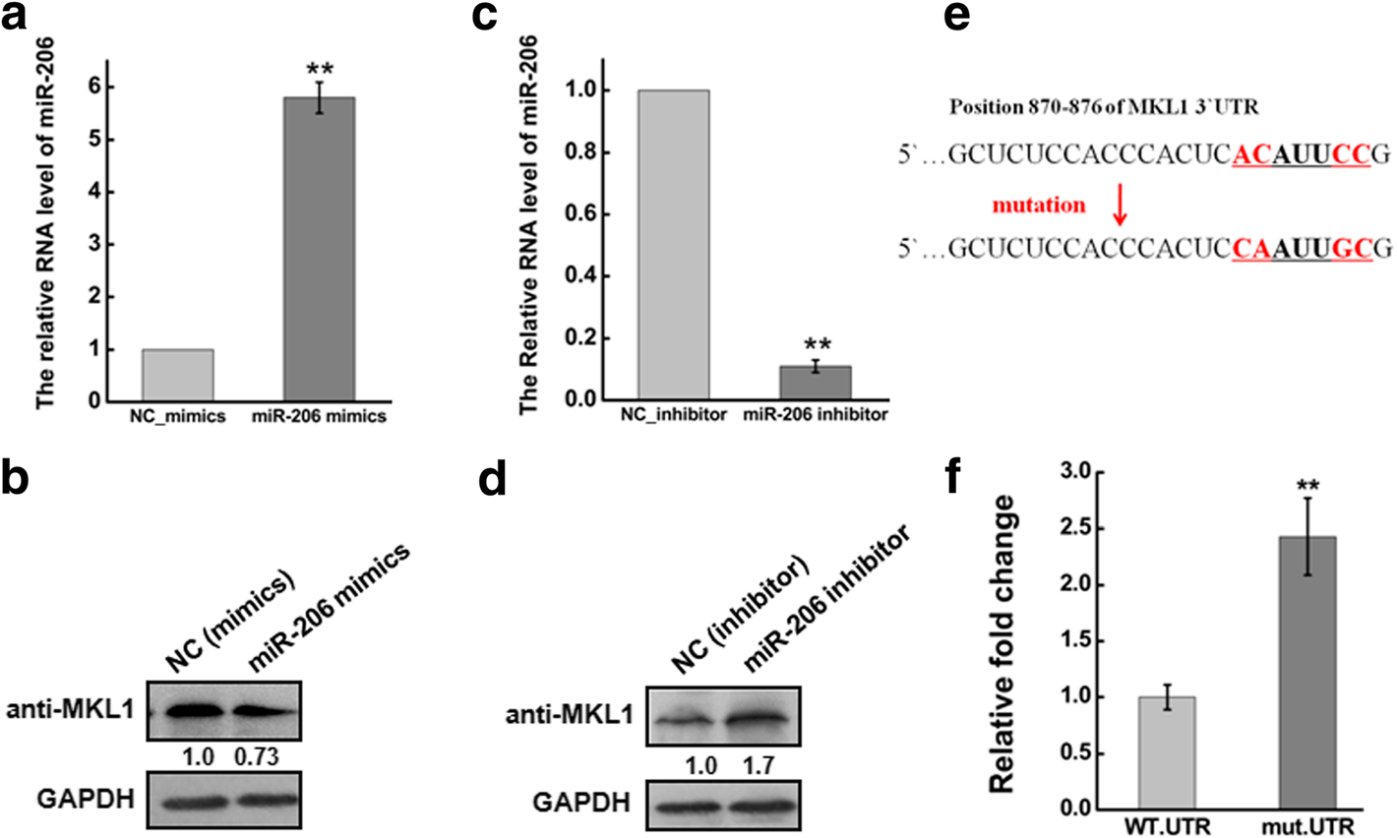
miR-206 degraded MKL1 by targeting the specific sites. **a** HeLa cells were transfected with miR-206 mimics or NC for 48 h. The abundance of miR-206 expression was determined by qRT-PCR. The expression level of miR-206 was normalized to U6. **b** Western blots analysis of the MKL1 protein expression at 48 h after transfected with miR-206 mimics or NC. GAPDH was used as an internal control. **c** qRT-PCR was used to measure the efficiency of miR-206 inhibition after transfecting miR-206 inhibitor at 48 h. The expression level of miR-206 was normalized to U6. **d** Western blots analysis of the MKL1 protein expression at 48 h after transfected with miR-206 inhibitor or NC. GAPDH was used as an internal control. **e** Diagram of MKL1–3’UTR containing reporter constructs. The seed sequence of miR-206 was underlined. Mutation contains 4-base-mutation at the miR-206 targeting region, abolishing its binding. **f.** In luciferase assays using HeLa cells, transfection of miR-206 and MKL1–3’UTR-mut increased the luciferase activities compared with transfection of miR-206 and MKL1–3’UTR-WT. Data are presented as means ± S.D. and results are from one representative experiment of at least three. *, *P <* 0.05; **, *P <* 0.01 (Student’s t test)

MicroRNA negatively regulated protein expression by targeting mRNA degradation or translation inhibition, which a class of small (~ 22 nt) non-coding RNA. Then we evaluated that whether MKL1 was regulated by defection of specific miRNA in cervical carcinoma. To investigate the targeting miRNA against MKL1, which involved in the modulation of cell migration and invasion, we used the two common prediction algorithms TargetScan [53] (http://www.targetscan.org/) and PicTar [54] (http://pictar.mdc-berlin.de/) to analyze 3’-UTR of MKL1. miR-206 could target 3’-UTR of MKL1 by two algorithms predicted. In order to further study that whether miR-206 expression was associated with migration and invasion of cancer cells, we transfected miR-206 mimics or inhibitor in HeLa cells using transfection reagent RNAi-mate. After transfected miR-206 mimics at 48 h, the RNA expression level of miR-206 was significantly increased **(**Fig. 5a**)**, and the protein MKL1 was obviously decreased **(**Fig. 5b**)**. However, the miR-206 inhibitor decreased the miR-206 RNA expression level **(**Fig. 5c**)** and increased MKL1 expression **(**Fig. 5d**)**. To further demonstrate the direct regulation of MKL1 by miR-206, we constructed luciferase reporters with the targets sequences of wile-type (WT-UTR) and mutated MKL1 3`-UTRs (mut-UTR)**.** In order to further identify that miR-206 targeted 3`-UTR of MKL1 by the predicted sites, we mutated 4 bases in the predicted sites **(**Fig. 5e**).** The result showed that the luciferase activities of MKL1-mut-luc were obviously increased, compared with negative control WT-UTR. All the data suggested that miR-206, as a potential targeting miRNA against MKL1, degraded MKL1 by targeting the specific sites in HeLa cells.

### Effect of miR-206 on cells migration and invasion in HeLa cells

MiR206, as a tumor suppressor, plays important roles in tumorigenesis and tumor progression of various human malignancies [55–57]. However, its involvement in cervical cancer has remained unclear. To investigate whether miR206 has a role in the mediating the biological roles between HOTAIR and MKL1, we firstly determined the functional roles of miR206 in the migration and invasion of HeLa cells.

**Fig. 6:**
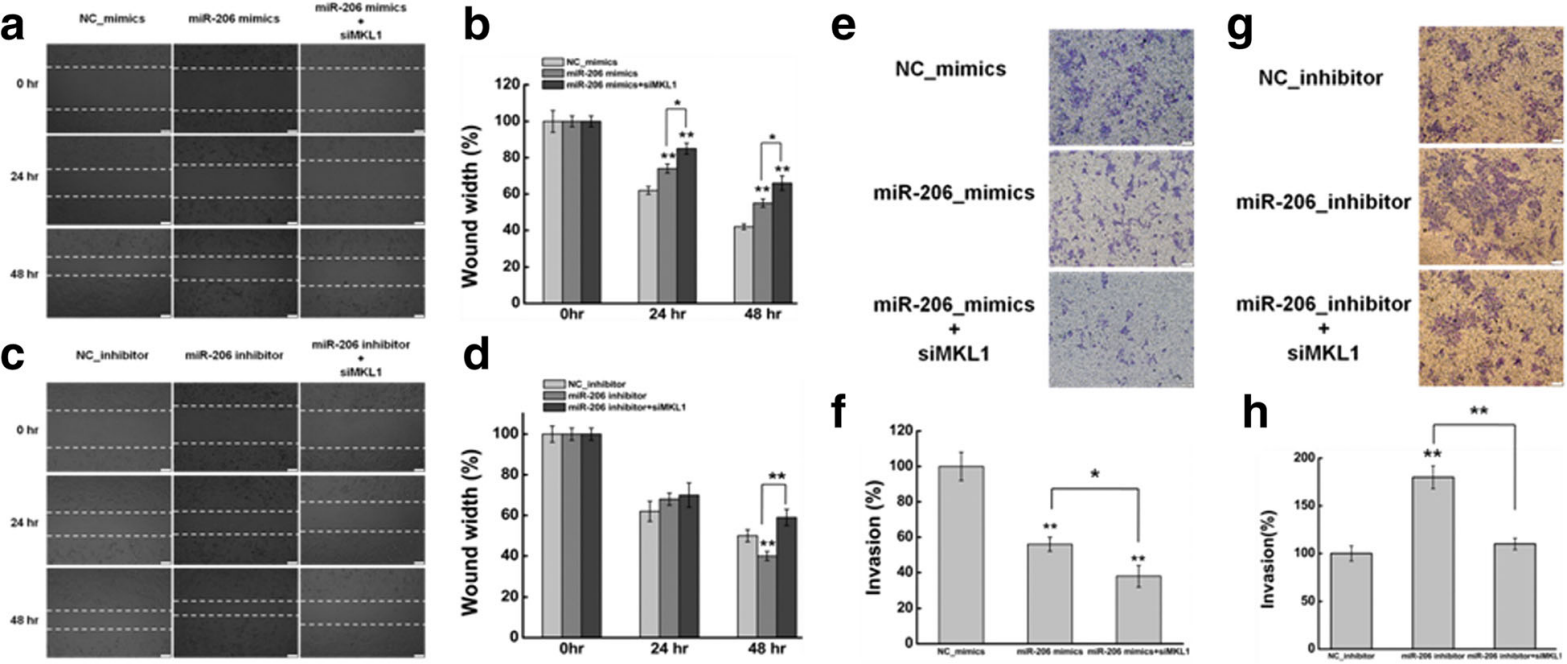
Effect of miR-206 on cells migration and invasion in HeLa cells. **a** The effect of expression miR-206 using miRNA mimics on cell migration was determined by wound healing assay. **b** Quantification of the wound healing assay. **c** The effect of inhibition miR-206 using miRNA inhibiton on cell migration was determined by wound healing assay. **d** Quantification of the wound healing assay. **e, g** The effect of miR-206 on cell invasion was determined in a Boyden chamber assay. **f, h** And the number of cells on the underside of the filter was determined and significantly (*P <* 0.05) changed invasion is indicated. Data are presented as means ± S.D. and represent results from three independent experiments. Statistically significant differences are indicated: *, P < 0.05; **, *P <* 0.01 (Student’s t test)

The effect of miR-206 on the migration of HeLa cells were evaluated by wound healing assay. Comparing with negative control, miR-206 mimics resulted in a significant decrease of cell migration in HeLa cells (Fig. 6a & b). Meanwhile, co-transfection of HeLa cells with miR-206 mimics and siRNA of MKL1 aggravating reduced effects of miR-206 mimics on cell migration compared with transfecting miR-206 mimics (Fig. 6a & b). In contrast, miR-206 inhibition increased the cell migration significant (Fig. 6c & d).What is more, co-transfection of HeLa cells with miR-206 inhibition and siMKL1 abrogated effects of miR-206 mimics on cell migration (Fig. 6c & d). Contrasted with miR-206 inhibition, the cells migration was significant reduced after co-transfecting miR-206 inhibition and siMKL1 (Fig. 6c & d).

To study the effect of miR-206 on the invasion of HeLa cells, we used the matrigel invasion assay. As shown in Fig. 6e & f, the number of invaded cells decreased significantly in miR-206 overexpression cells by transfecting miR-206 mimics in comparison with the negative control. And co-transfected miR-206 mimics and siMKL1 resulted in the reduced invasion capability significantly compared with miR-206 mimics (Fig. 6e & f). In contrast, inhibition miR-206 increased the capability of invasiveness in HeLa cells in comparing with negative control (Fig. 6g & h). Meanwhile, the number of invaded cells decreased significantly after co-transfecting miR-206 inhibitor and siMKL1, compared with miR-206 inhibition cells (Fig. 6g & h). Contrasted with miR-206 inhibitor, co-transfected miR-206 inhibitor and siMKL1 reversed the effect of miR-206 inhibition on cell invasion.

Based on these results, we proposed that miR-206 inhibited cell migration and invasion in HeLa cells, and MKL1 could partially abrogate these effects of miR-206.

### HOTAIR regulated expression of miR-206

**Fig. 7:**
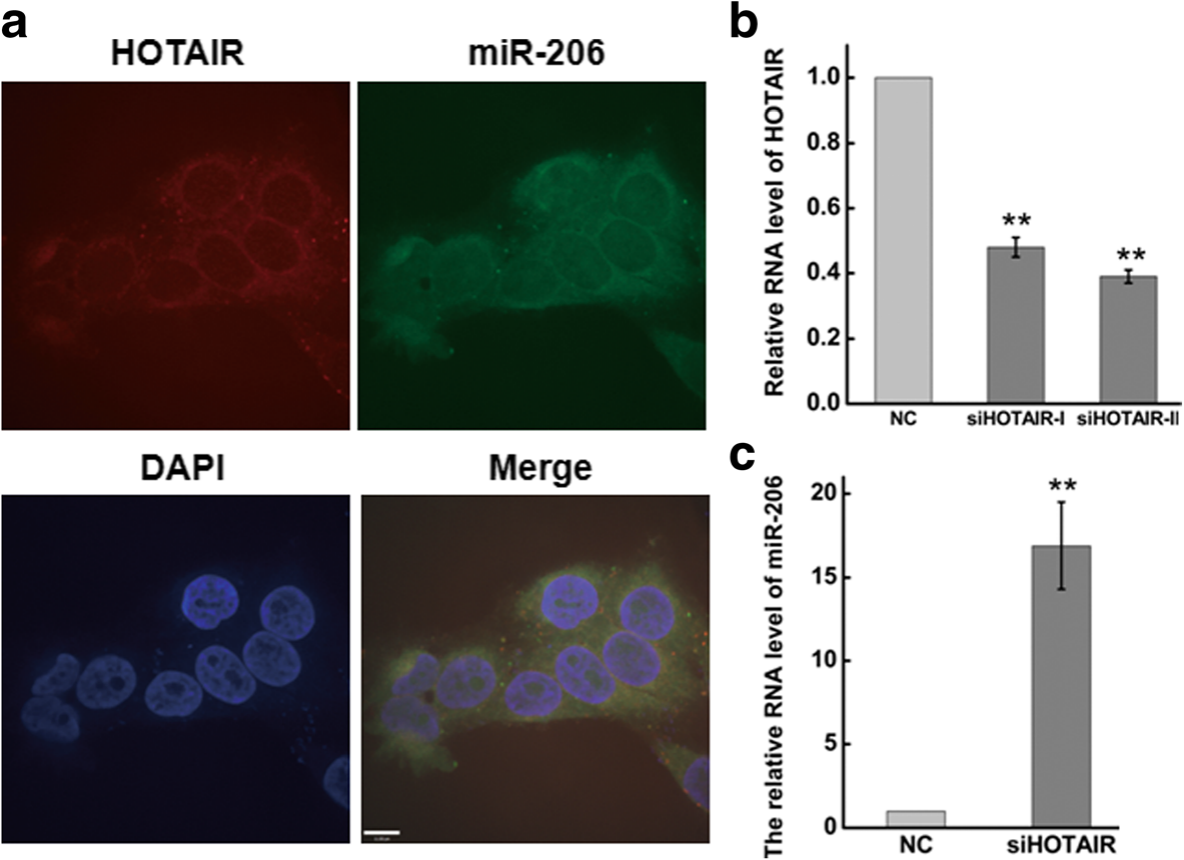
Detect the localization of HOTAIR and miR-206 by RNA FISH in HeLa cells. **a** Detect the localization of HOTAIR and miR-206 by RNA FISH in HeLa cells. The HOTAIR probes were labelled with Alexa Fluor 647 at the 5 terminal. And the probe of miR-206 was labelled with FAM. Nuclei are stained blue with DAPI. **b** The efficiency of HOTAIR knockdown was determined after transfecting siRNA targeted against HOTAIR in HeLa cells at 48 h by qRT-PCR. The expression level of HOTAIR was normalized to GAPDH. HeLa cells were transfected with siHOTAIR or siNC for 48 h. **c.** The RNA expression of miR-206 after transfecting siHOTAIR at 48 h by qRT-PCR. The expression level of miR-206 was normalized to U6. Data are presented as means ± S.D. and results are from one representative experiment of at least three. *, *P* < 0.05; **, *P* < 0.01 (Student’s t test)

In this study, we have demonstrated that the protein expression of MKL1 was increased after inhibition HOTAIR, and miR-206 also mediated the expression of MKL1. Whether there was a relationship between HOTAIR and miR206 remained to be explored. To investigate the relation between HOTAIR and miR-206, we firstly detected the distribution of HOTAIR and miR206 using RNA-fluorescence in situ hybridization (RNA-FISH) technology, ensuring the expression abundance. RNA-FISH, as an indispensable tool for the detection and localization of RNA, was used to study the distribution of HOTAIR and miR-206. As shown in Fig. 7a, the majority of the HOTAIR or miR-206 expression were detected in cytoplasm in HeLa cells using the junction probes.

Knockdown HOTAIR by transfecting siRNA against HOTAIR and the knockdown efficiency was evaluated by qRT-PCR. As shown in Fig. 7b, the abundance of HOTAIR in HeLa cells reduced more than 60% at 48 h. And the RNA expression level of miR-206 exhibited a dramatic decrease at 48 h after HOTAIR inhibition **(**Fig. 7c**)**. This data indicated HOTAIR could be a potential negative regulator of miR-206 in HeLa cells.

### HOTAIR affects MKL1 distribution in HeLa cells

**Fig. 8:**
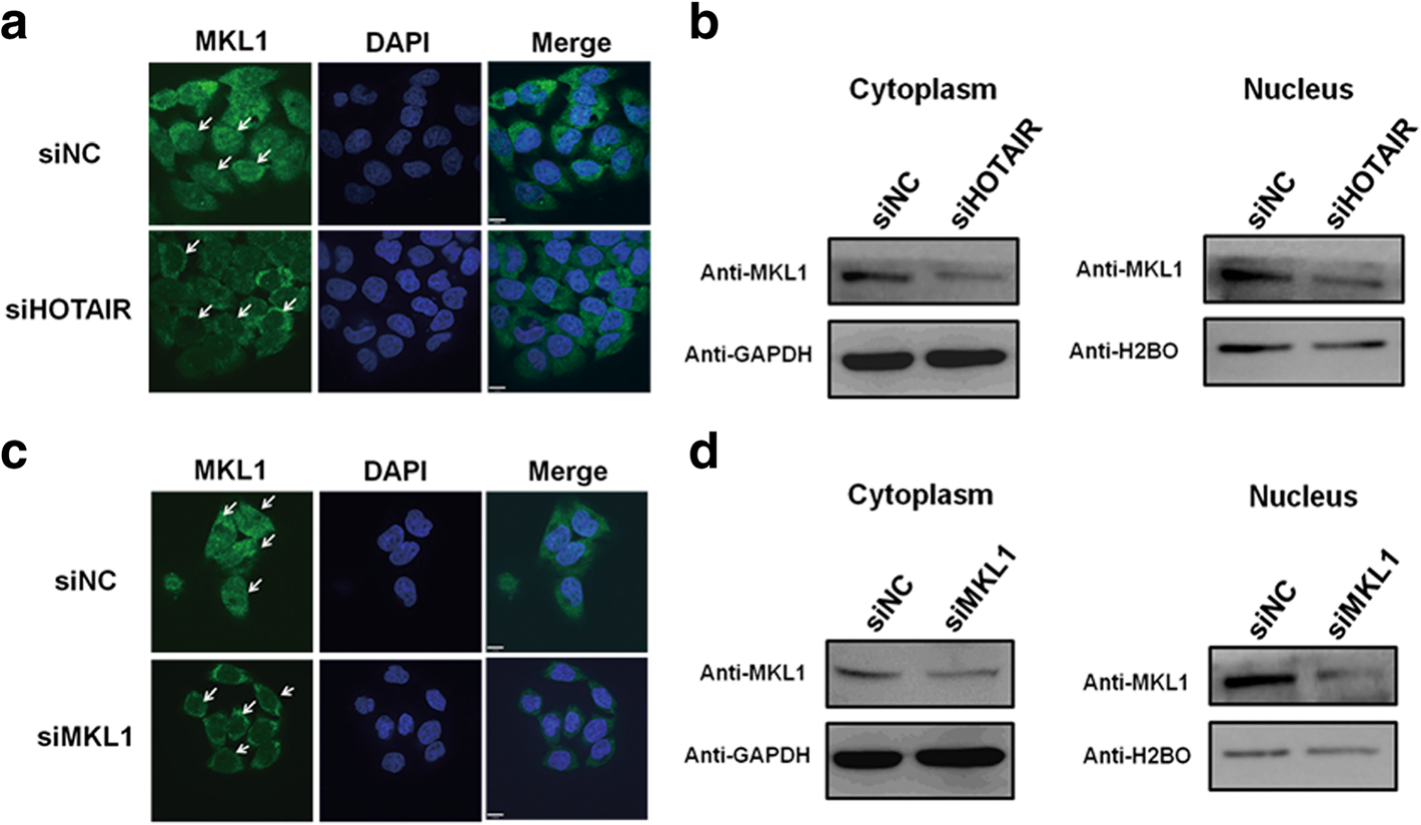
Inhibition of HOTAIR affects the location of MKL1 **a.** Representative images showing the distribution of MKL1 in HeLa cells after HOTAIR knockdown under confocal microscopy. **b** Western blotting analysis the expression of MKL1 in cytoplasm and nucleus after HOTAIR inhibition. **c** Representative images showing the distribution of MKL1 in HeLa cells after MKL1 knockdown under confocal microscopy. **d** Western blotting analysis the expression of MKL1 in cytoplasm and nucleus after transfecting siRNA targeted against MKL1. Data are presented as means ± S.D. and results are from one representative experiment of at least three. *, *P* < 0.05; **, *P* < 0.01 (Student’s t test)

As the co-activators of SRF known, MKL1 continuously shuttles between the nucleus and cytoplasm and may help transducer signals from the cytoskeleton to the nucleus [35]. Nuclear accumulation of MKL1 will increase the migration and invasion of cancer cells [58]. To determine whether HOTAIR knockdown can affect the subcellular localization of MKL1, we compared the MKL1 distribution before and after HOTAIR inhibition. As shown in Fig. 8a, HOTAIR knockdown resulted in a marked reduced in the nucleus of the distribution of MKL1. The protein expression level of MKL1 was significantly decreased in cytoplasm and nucleus after HOTAIR inhibition by western blotting **(**Fig. 8b**)**. Meanwhile, the distribution of MKL1 was changed after MKL1 knockdown, and the cytoplasm nucleus MKL1 were obviously decreased compared with the cells transfected with the control siRNA **(**Fig. 8c & d**)**. These results showed that inhibition of HOTAIR expression can change the subcellular localization of MKL1, which support the notion that the ability of migration and invasiveness were decreased after HOTAIR knockdown, at least in part, through the change of MKL1 distribution in HeLa cells.

### MKL1 activated HOTAIR transcription by binding CArG box

As the co-activators of the transcription factor SRF, MKL1 regulated various gene transcription, which contains a core sequence known as the CArG-box (CC(A/T)_6_GG) in the promoter regions. To determine that how transcription of HOTAIR was regulated, we the performed a search for the potential CArG-box binding sites using software programs. MatInspector (www.genomatix.de/online_help/help_matinspector/matinspector_help.html) and TFSEARCH (www.cbrc.jp/research/db/TFSEARCH.html) in the promoter region of HOTAIR and found that one putative CArG-box element at ~ 524 upstream of HOTAIR which could be recognized by MKL1/SRF.

**Fig. 9:**
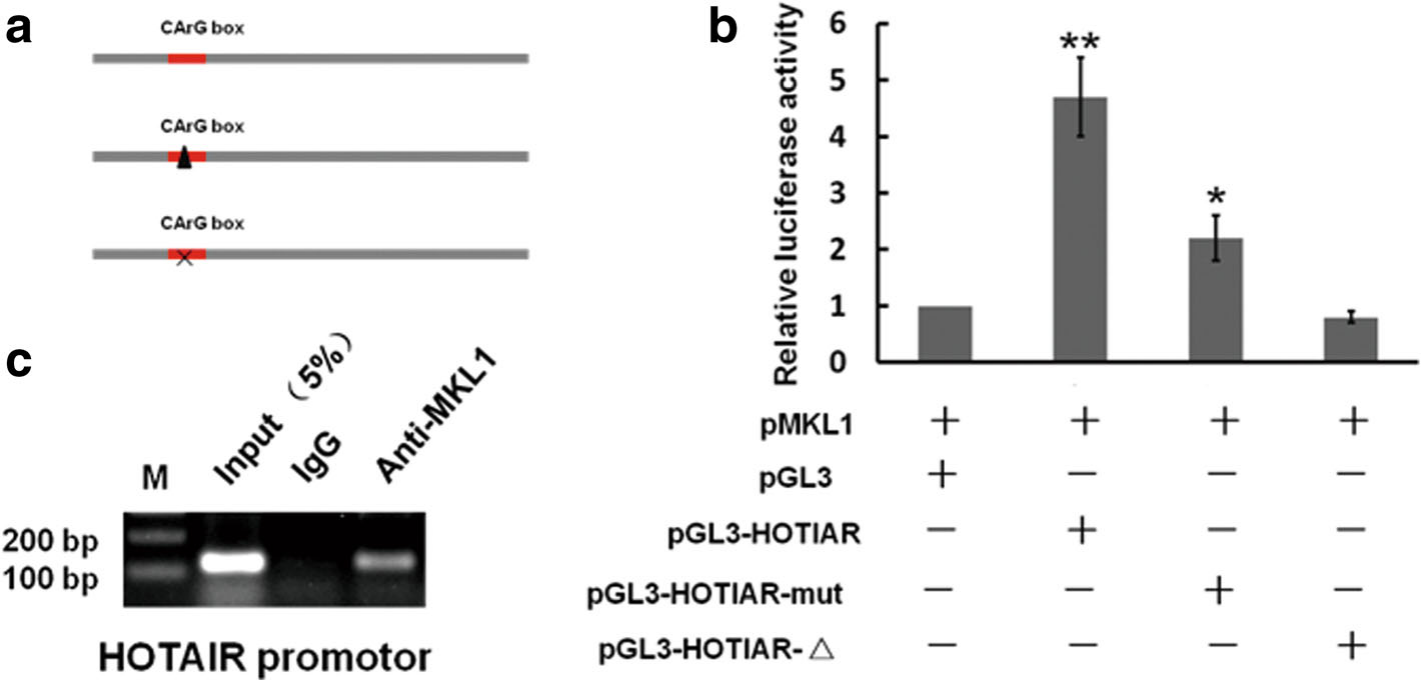
MKL1 activated HOTAIR transcription by binding the CArG box in promoter region. **a** Diagram of the CArG box in HOTAIR promoter region. **b** Cells were transfected with luciferase vectors containing the promoter region of HOTAIR, along with MKL1 expression vector. Luciferase activity was measured at 48 h after transfection. HOTAIR promoter activity was decreased after transfecting with plasmids, which mutated or deleted CArG box in the promoter region of HOTAIR, compared with the control. Data are presented as means ± S.D. and results are from one representative experiment of at least three. *, *P* < 0.05; **, *P* < 0.01 (Student’s t test). **c** Chromatin immunoprecipitation (ChIP) assay was used to determined the interaction between HOTAIR and MKL1 in COS7 cells. Input = 5% of total lysate. IgG = immunoglobulin G

To evaluated whether MKL1/SRF regulated the expression of HOTAIR, we firstly cloned the fragment of HOTAIR promoter region from the genome of HeLa cells, and inserted into the pGL3 basic firefly luciferase reporter (Promega) and co-transfected the plasmid pCDNA3.1-MKL1 into Cos-7 cells, which eliminated the endogenous MKL1 expression. Then, we mutated or deleted the CArG-box, as depicted in Fig. 9a, and co-transfected with pCDNA3.1-MKL1. The results showed that deletion or mutation of the CArG-box element abolished the effects of MKL1/SRF on the promoter activity of HOTAIR **(**Fig. 9b & c). This data suggested that MKL1/SRF may affect the transcription activity of HOTAIR through binding the CArG-box. To further tested the mechanism of this regulation, we used chromatin immunoprecipitation (ChIP) to investigate in HeLa cells. As shown in Fig. 9d**,** MKL1was highly enriched in the DNA fragments compared with negative control IgG immunoprecipitates. Based on these results, we speculated that MKL1 activated HOTAIR transcription level through binding the CArG-box element via the serum response factor SRF.

## Discussion

As a new class of non-coding RNAs, lncRNAs have emerged as a new and crucial layer of gene regulation (13–15). lncRNA are critical for genome organization and regulation of multiple gene expression and have essential roles in tumorigenesis by influencing the complex networks involving proteins and other types of ncRNAs [44–46, 59]. Among them, HOTAIR is one of the few well-studied lncRNAs and has emerged as a key regulator of carcinogenesis and metastasis, and a potential prognostic marker [15, 19, 60]. Considerable attention has thus been given to determining its functions as well as to identifying its target genes. Previous studies have shown that HOTAIR can regulate diverse biological processes by modulating the RNA or protein levels of hundreds of genes [38, 45]. To well understanding the precise molecular mechanism underlying the potential role of HOTAIR in cancer cells, it is essential to investigate the relationship between HOTAIR and the target genes.

As a transcription co-activator factor, MKL1 is broadly expressed in many tissues [33], and regulate the expression of signalling molecules, transcriptional factors and numerous cytoskeletal components, including actin genes, non-muscle myosins and vinculin through SRF [33, 52, 58, 61–64]. MKL1 has been implicated in cancer progression and metastasis, such as thyroid cancer [65], breast cancer [66] and liver cancer [67]. Knockdown of MKL1 could reduce the adhesion, migration and invasion of cancer cells [68]. Meanwhile, MKL1 double knockdown in mouse embryonic fibroblasts (MEFs) impairs cell migration, consistent with an important role for SRF and MKL1 in cytoskeletal organisation and actin homeostasis [68]. By contrast, MKL1 induced sarcoma cell differentiation, blocks malignant growth and acts as a tumor suppressor [61]. There is growing evidence of an association between the expression of MKL1 and tumor invasiveness and aggressiveness [69]. Therefore, MKL1 is emerging as an indicator of poor prognosis for cancer patients and an attractive potential target for cancer therapy [70]. In agreement with published data, we found that MKL1 knockdown led to decreased invasion and migration in HeLa cells. While, overexpressed MKL1 dominantly increased the ability of migration and invasiveness. As shown in Fig. 7, we further informed that the expression of HOTAIR and MKL1 were positively correlation in cervical cancer patient. By investigating the impact of HOTAIR inhibition and MKL1 knockdown in HeLa cells, we have shown that the effects of the migration and invasion in cancer cells of HOTAIR knockdown can be mimicked by the respective manipulation of MKL1 expression. Importantly, the ability of migration and invasiveness in HeLa cells were significantly decreased after HOTAIR inhibition and MKL1 knockdown simultaneously.

MiRNAs are highly conserved, single stranded, endogenous non-coding RNAs of 19–25 nt in length found in diverse organisms [71], which can down regulate various gene products by repressing translation or cleaving RNA transcripts in a sequence-specific manner [72–75]. MiR206, as a tumor suppressor, regulated several oncogenes expression in various types of tumor cancers. And, the expression of miR206 were significantly down-regulated in cervical cancer, compared with adjacent normal tissues [76]. In this study, we further confirmed that knockdown HOTAIR could enhance miR206 RNA expression level in HeLa cells. Our results showed that increased miR206 expression could significantly slow down the migration and invasion in HeLa cells. To the contrary, the effects were enhanced after transfecting miR206 inhibition. Furthermore, MKL1 was down-regulated by miR206 via targeting its 3-UTR, as a potential target gene of miR206 in HeLa cells. Together, these data suggest that HOTAIR exerts its effects on migration and invasion of cancer cells, at least in part, through the regulation of MKL1 via inhibition of miR206 expression in HeLa cells.

The functional studies also demonstrated that HOTAIR could affect the distribution of MKL1 in HeLa cells. MKL1 plays an important role in a number of biological processes, such as cell growth, cell migration and organization of the cytoskeleton [67, 77]. It continuously shuttles between the nucleus and cytoplasm to transduce signals [35]. And when it accumulates in the nucleus, MKL1 activated targeting gene transcription through SRF [67]. Extracellular signal-regulated kinase 1/2 (ERK1/2) could phosphorylate MKL1 at S454 and increase its nucleus exports [78]. Previously researches reported that HOTAIR may indirectly increase the expression of SETDB1 [79]. As a histone methyltransferaes, SETDB1 is known to repress gene expression in euchromatin through H3K9 trimethylation [80, 81], and directly decreased ERK1/2 activity [82]. In this study, we found that HOTAIR inhibition led to the change of the distribution of MKL1 in HeLa cells, which the expression of nuclear localization of MKL1 was dramatically decreased compared with in cytoplasm. These results suggested that HOTAIR exerts its effects on migration and invasion of cancer cells, through the change of the localization of MKL1 in HeLa cells through MKL1 phosphorylation via ERK1/2 pathway.

Based on our data, we propose a model depicting the molecular mechanism of HOTAIR in regulating migration and invasion of cancer cells. We suggested that HOTAIR promotes cell migration and invasion of cancer cells through at least three mechanisms. The first mechanism is that HOTAIR serves as a molecular scaffold to link PRC2 and LSD1/CoREST/REST protein complexes to regulate hundreds of genes. The second mechanism is that HOTAIR regulated MKL1 expression via inhibition of miR206 expression. As a transcription activator factor, the distribution of MKL1 caused by HOTAIR inhibition may change the expression of MKL1 in cytoplasm and nuclear and the change of the distribution of MKL1 can be another mechanism of decreased cell migration and invasion. The combination of all three mechanisms promotes cell migration and invasion in cancer cells, While MKL1 may be a vital molecule in HOTAIR-mediated oncogenic signaling.

It is highly interestingly that we found that predicted a putative binding site of MKL1 in the promoter region of HOTAIR. MKL1 forms a complex with SRF [33], and binds to DNA sequence elements called CArG box to allow for target gene transcription [63]. In this study, we demonstrated that MKL1 induced HOTAIR transcription through direct interacted with the CArG box in the HOTAIR promoter region.

## Conclusion

In conclusion, we have performed that HOTAIR exerts its effects on migration and invasion of cancer cells, at least in part, through the regulation of MKL1 expression via inhibition of miR206 expression. The elucidation of the role of MKL1 may shed light into the molecular basis of the HOTAIR induced tumor metastasis. It is now important to further characterize the interactions between HOTAIR and MKL1, which would contribute to understand the development of cancer.

## Additional files

Additional file 1: Table S1. The list of primers sequences. (DOCX 29 kb)

Additional file 2: Figure S1. The effects on migration and invasion of MKL1 KO cells lines via CRISPR/Cas 9 system. **(A).** The MKL1 expression level was determined by western blot in MKL1 KO cells. GAPDH serves as the loading control. **(B).** Wound healing assay the effect on cell migration was determined after MKL1 depletion. **(C).** Quantification of the wound healing assay. **(D).** The effect on cell invasion was determined after MKL1 depletion in a Boyden chamber assay. **(E).** The number of cells on the underside of the filter was determined and significantly (*P* < 0.05) changed invasion is indicated. Data are presented as means ± S.D. and results are from one representative experiment of at least three. *, *P* < 0.05; **, *P* < 0.01 (Student’s t test). (ZIP 4524 kb)

Additional file 3: Figure S2. Cervical cancer tissue microarray analysis. (A). Hematoxylin and eosin (HE)-stained cervical cancer tissues and adjacent normal tissues. **(B).** HOTAIR was stained by in situ hybridization; high level of HOTAIR expression in cervical cancer tissues compared with adjacent normal tissues. (C). The expression of MKL1 were detected in cervical cancer tissues and adjacent normal tissues by IHC. The MKL1 expression almost was enhanced in cervical cancer tissues compared with adjacent normal tissues. The images were acquired by Pannoramic MIDI with a 20 × microscope objective. (ZIP 10865 kb)

## Abbreviation

CHIP: Chromatin Immunoprecipitation Assay
DAPI: 4′6-diamidino-2-phenylindole
ERK1/2: Extracellular signal-regulated kinase 1/2
HE: Hematoxylin and Eosin staining
HOTAIR: HoxC transcript antisense intergenic RNA
IHC: Immunohistochemistry
ISH: In situ hybridization
lncRNA: Long non-coding RNA
MKL1: Megakaryoblastic leukemia 1
MRTF: Myocardin related transcription factor
PRC2: Polycomb repressive complex 2
qPCR: quantitative Polymerase Chain Reaction
RNA-FISH: RNA-fluorescence in situ hybridization
RNAi: RNA interference
SRF: Serum response factor
