# Supplementary material for: LncRNA HOTAIR promotes cell migration and invasion by regulating MKL1 via inhibition miR206 expression in HeLa cells
Source: Cell Commun Signal. 2018 Feb 1;16:5. doi: 10.1186/s12964-018-0216-3 (PMC5796349; doi:10.1186/s12964-018-0216-3)
Supplement: Supplementary file 1 — The list of primers sequences. (DOCX 29 kb) [file 12964_2018_216_MOESM1_ESM.docx]

| **HOTAIR** | siHOTAIR-I | 5’-GAACGGGAGUACAGAGAGAUU-3’ |
| --- | --- | --- |
|  | siHOTAIR-II | 5’-CCACAUGAACGCCCAGAGAUU-3’ |
| **MKL1** | siVASP-I | 5’-GCUGAAGAGAGCCAGACUATT-3’ |
|  | siMKL1-II | 5’-GCCUGAAGGAAGCCAUCAUTT-3’ |
|  | siMKL1-III | 5’-CCAAGGAGCUGAAGCCAAATT-3’ |
| **Negative control** | siNC | 5’-UUCUCCGAACGUGUCACGUTT-3’ |
| **miR-206** | Mimics_NC | 5’-UUCUCCGAACGUGUCACGUTT-3’ |
| **miR-206** | Mimics | 5’-UGGAAUGUAAGGAAGUGUGUGG-3’ |
| **miR-206** | Inhibitor_NC | 5’-CAGUACUUUUGUGUAGUGCAA-3’ |
| **miR-206** | Inhibitor | 5’-CCACACACUUCCUUACAUUCCA-3’ |

**Table S1-1: List of siRNA sequences**

**Table S1-2: Primer sequences for qRT-PCR**

| **HOTAIR** | Forward | 5’-GGCAGCACAGAGCAACTCTA-3’ |
| --- | --- | --- |
|  | Reverse | 5’-GAGTGCAAAG TCCCGTTTG-3’ |
| **GAPDH** | Forward | 5’-CCGGGAAACTGTGGCGTGATGG-3’ |
|  | Reverse | 5’-AGGTGGAGGAGTGGGTGTCGCTGTT-3’ |
| **MKL1** | Forward | 5’-GTCTCGGGCACCAAAACTGAGC-3’ |
|  | Reverse | 5’-TGGAGCCAGGCCTGCTGC-3’ |
| **miR-206** | RT Primer | 5’-GTCGTATCCAGTGCAGGGTCCGAGGTATTCGCACTGGATACGACCCACAC-3’ |
| **U6** | RT Primer | 5’-GTCGTATCCAGTGCAGGGTCCGAGGTATTCGCACTGGATACGACAAAAATATG-3’ |
| **miR-206** | Forward | 5’-GCCCGCtggaatgtaaggaagt-3’ |
|  | Reverse | 5’-CCAGTGCAGGGTCCGAGGT-3’ |
| **U6** | Forward | 5’-GCGCGTCGTGAAGCGTTC-3’ |
|  | Reverse | 5’-CCAGTGCAGGGTCCGAGGT-3’ |

**Table S1-3****: Primer sequences of MKL1**

| **MKL1** | PF-1 | ATGCCGCCTTTGAAAAGTCCAGC |
| --- | --- | --- |
|  | PR-1 | CTACAAGCAGGAATCCCAGTGCAGCT |
| **MKL1** | PF-2 | GCT**GGATCC**ATGCCGCCTTTGAAAAGTCCAGC |
|  | PR-2 | GTC**CTCGAG**CTACAAGCAGGAATCCCAGTGCAGCT |

**Table S1-4: The sequences of gRNA targeting MKL1**

| **MKL1** | gRNA 1 | GGCCAAGGAGCTGAAGCCAAAGG |
| --- | --- | --- |
|  | gRNA 2 | GGTTG TGTCTCAACTTCCGATGG |
|  | gRNA 3 | GGATAGTGGTTCCATTGGTGAGG |
|  | gRNA 4 | GGACTTGGCAGTGGGGATA GTGG |

**Table S1-5: Primer sequences of MKL1 for Luciferase assay**

| **MKL1** | PF-3 | gctGCTAGC CTCTCTGGCTCAAGACGGGGTG |
| --- | --- | --- |
|  | PR-3 | gtcGCTAGC GTTCACACACCTTCCCCATCTTTGT |
| **MKL1** | PF-4 | ACCCACTCCAATTGCGTCCCC |
|  | PR-4 | ACGCAATTGGAGTGGGTGGAGAG |

**Table S1-6: Primer sequences of HOTAIR for Luciferase assay**

| **HOTAIR promoter** | PF1 | gct GGTACC AAGCGGAGAGAGTCCCACACATG |
| --- | --- | --- |
|  | PR1 | gtc CTCGAG ACAGGCGAGTCAGAGTTCCCCAC |
| **HOTAIR promoter** | △PF | ACCCACTCCAATTGCGTCCCC |
|  | △PR | ACGCAATTGGAGTGGGTGGAGAG |
| **HOTAIR promoter** | Mut PF | TTCCCCGGCCGGCCCTATTTTACGAGC |
|  | Mut PR | TAGGGCCGGCCGGGGAAGAAATAGGGTC |

**Table S1-7: Primer sequences of MKL1 for ChIP**

| **MKL1** | PF-5 | AGCAACCCGACCCTAT |
| --- | --- | --- |
|  | PR-5 | GCAGCGGCTCCATTTCC |

**Table S1-8: Sequences of HOTAIR probes for RNA FISH**

| **HOTAIR** | HOTAIR 1 | 5-GATCAAGCTCCAGAGCACAG-3 |
| --- | --- | --- |
|  | HOTAIR 2 | 5-CAGGACCTTTCTGATTGAGA-3 |
|  | HOTAIR 3 | 5-TACTTATAAGGAAGGCGCCG-3 |
|  | HOTAIR 4 | 5-CACGTTTGTTCCGGGAACTG-3 |
|  | HOTAIR 5 | 5-GTCTTGTTAACAAGCCTCAT-3 |
|  | HOTAIR 6 | 5-AAATCCAGAACCCTCTGACA-3 |
|  | HOTAIR 7 | 5-AAATTCCGGAGCAGCTCAAG-3 |
|  | HOTAIR 8 | 5-GATGCATTCTTCTAGACCTA-3 |
|  | HOTAIR 9 | 5-TTCAGGCATTGGGAATGGTA-3 |
|  | HOTAIR 10 | 5-TACCTACCCAATGTATGGAA-3 |

**Table S1-9: Sequences of miR-206 probes for RNA FISH**

| miR-206 5’ FAM -ccacacacuuccuuacauucca 3’ |
| --- |
